# The InterHerz project - a web-based psychological treatment for cardiac patients with depression: study protocol of a randomized controlled trial

**DOI:** 10.1186/1745-6215-13-245

**Published:** 2012-12-28

**Authors:** Nadine Messerli-Bürgy, Jürgen Barth, Thomas Berger

**Affiliations:** 1 Department of Clinical Psychology and Psychotherapy, University of Bern, Gesellschaftsstrasse 49, Bern, 3012, Switzerland; 2 Institute of Social and Preventive Medicine (ISPM), University of Bern, Niesenweg 6, Bern, 3012, Switzerland

**Keywords:** Psychological treatment, Web-based, Depression, Guided self-help, Heart disease, Randomized controlled trial

## Abstract

**Background:**

Patients with heart disease often suffer from difficulties in psychological adaptation during cardiac rehabilitation. Mood disorders such as depression are known to be highly prevalent in cardiac patients and to have a negative impact on the progression of coronary heart disease. However, cardiac patients have difficulties to get psychological treatments due to low availability and motivational difficulties. Web-based interventions have been proven to be effective in treating depressive symptoms. Deprexis is a promising web-based psychological treatment which was devised for depressed patients. The aim of the study InterHerz is to examine if Deprexis is an effective psychological treatment to reduce stress and depression in cardiac patients.

**Methods/Design:**

The sample will consist of 80 depressed patients randomized to an intervention group or a waitlist (10 weeks). Patients are recruited via cardiologists, cardiac rehabilitation units and the website of the Swiss Heart Foundation. Patients have access to a guided self-help program in which they work themselves through several modules and receive feedback from a clinical psychologist. Pre- and post-assessments, and a six-month follow-up, are conducted using online questionnaires and diagnostic interviews.

**Discussion:**

Deprexis is a new web-based treatment which has the potential to help depressed cardiac patients with limited access to psychological treatment to increase their mental health.

**Trial registration:**

Current Controlled Trials ISRCTN45945396

## Background

There is a large body of evidence that high or prolonged stress, depression and low social support are predictive of coronary artery health [1-4]. In particular, depression has been identified as a psychosocial factor with enormous potential to influence the course and outcome of coronary artery disease [3,5,6]. Despite the empirical evidence that depression increases the risk of cardiovascular morbidity and mortality itself, there is no common accepted model that describes the underlying mechanisms [7,8]. Both direct and indirect pathways have been put forward. On one hand, direct influences of depression on physiological factors may lead to atherosclerosis or coronary events. On the other hand, depression leads to an increase in coronary risk factors (that is, inflammation), which in turn may cause heart disease. Finally, there may be some underlying factors (that is, distress, personality, social environment and health behavior) influencing the risk for both depression and heart disease [9]. Psychosocial and behavioral factors correlate both with depression and heart disease. Depression is associated with poor health behavior, maladaptive coping style, social isolation, and chronic life stress [10]. Behavioral risk factors, such as smoking, low physical activity, a poor diet, and the failure to adhere to medical recommendations mediate the relationship of depressive disorders with heart disease [11,12].

Several studies have focused on the treatment of depressive symptoms in cardiac patients. The study, enhancing recovery in coronary heart disease patients with depres-sion (ENRICHD), examined the efficacy of psychotherapy (cognitive-behavioral approach) on depression [13]. The effect size (ES) of short-term efficacy in comparison to the control group were small (ES = 0.35), and disappeared on long-term follow-up. Furthermore, the Canadian cardiac randomized evaluation of antidepressant and psychotherapy efficacy (CREATE) study, investigating the benefit of an interpersonal psychotherapy (IPT) [14], only detected a tendency towards a reduced depression level in the IPT group compared to the non-treated group (ES = 0.20). Larger ES over 0.70 were reported for psychotherapy in depressed but otherwise healthy people. However, these effects might be overestimated due to publication bias [15].

One of the greatest challenges of a psychotherapeutic approach to support treatment success of cardiac patients with depression is the time and resource intensity of most traditional approaches of treatment delivery. The opportunity to develop new forms of intervention, for example, web-based treatment, has never been more feasible. To date, there is sparse evidence that psychotherapy influences the progress of heart disease.

Many people are now web-literate and many have access to the Internet. Today, internet-based technologies are used at almost all levels of psychosocial services. Apart from possible cost-effectiveness and the fact that web-based interventions can flexibly be used independently of time and place, a major advantage of the web-based approach is that it offers support to people who may not otherwise seek or reach treatment.

Over recent years, web-based treatments have been used with success to promote health [16,17] and have shown promising outcomes for several psychiatric conditions, including depression [18-20]. Particularly web-based guided self-help approaches, in which the presentation of a web-based self-help program is combined with minimal but regular therapist contact via e-mail, have shown promising results. Emerging evidence from meta-analyses [19,21] and from a reanalysis of data across trials [22] suggests a superiority of guided versus unguided self-help, both in terms of efficacy and drop-out rates.

There are only a few web-based interventions for cardiac patients that have been developed and evaluated so far [23]. Preliminary results confirm that a web-based intervention for cardiac patients can help to change psychosocial risk factors. Web-based interventions can reduce symptoms of depression [24,25] and anxiety [25,26] and help to increase social support [27]. Besides this, cardiac patients report improvements in managing their physical condition by knowing more about their disease, and therefore increase their perception of control [28] and change their health behavior [29,30]. In addition, improvements in quality of life [27,31,32], and even improvements in adherence due to the presentation of a cardiac-specific information tool [25,33] have been reported.

There are only two web-based psychological treatment programs to have been developed so far: a specific web-based distress management program for patients with cardioverter defibrillator [34] and a web-based treatment for cardiac patients with depression [35].

In consideration of the difficulties of treating cardiac patients, this intervention study InterHerz strikes a new path by implementing a proven and promising web-based approach called Deprexis in a somatically ill patient group with depression [36-38]. Deprexis has been developed to reduce depressive symptoms in physically healthy patients with a depressive disorder. To our knowledge, this is the only psychological treatment for German-speaking patients with depression, and in contrast to other web-based tools, it integrates different psychotherapeutic approaches [36].

## Methods/Design

### Aims

The primary aim of InterHerz is to investigate whether the proven web-based treatment Deprexis is effective to reduce depression and perceived stress in cardiac patients. Secondary aims are to examine the mid-term effects of Deprexis on depression, perception of stress, social support, and changes in health behavior after six months follow-up.

### Design

The study uses a randomized controlled design comparing a web-based treatment with a waitlist control condition. All patients in the treatment group receive basic information on the use of the program, a booklet on depression and heart disease, a personal user name and a password. They have immediate access to the program. Patients under the waitlist condition receive the equivalent access to the program after a waiting period of 10 weeks. The randomization sequence is generated by the website Research Randomizer ([http://www.randomizer.org]) and concealed in the study center of the Institute for Social and Preventive Medicine, University of Bern (Switzerland).

### Sample size calculation

In a previous study testing the same psychological treatment in depressed patients, medium to large between-group effect sizes were found [37]. Assuming medium between-group effects, 80 randomized patients in total (40 patients per treatment condition) are needed in this study. This estimation is based on the assumptions of a level of significance of 0.05 (two-sided), 80% statistical power (1-Beta), and correlations between pre- and post-assessment found in previous studies, and the estimate was calculated using G-Power [39].

### Study sample

**Figure 1:**
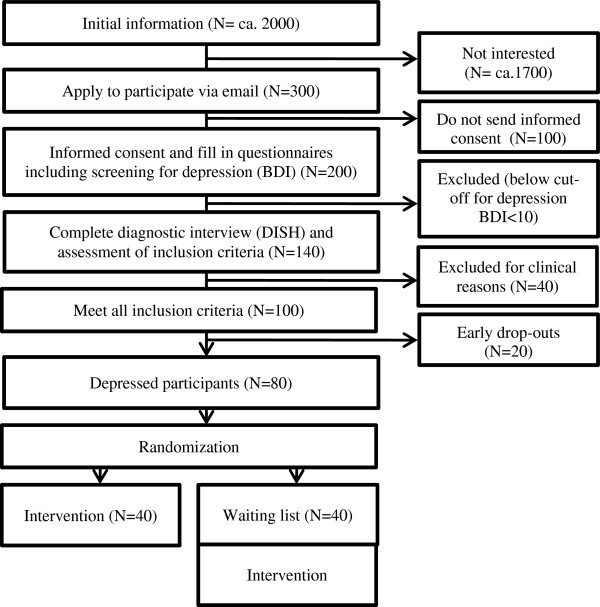
Recruitment and participant flow.

Patients with any coronary artery disease are invited to participate in the study. We estimate a number of more than 2,000 patients who shall be reached through the collaborating cardiology institutions (for example, rehabilitation centers, hospitals). The Swiss Heart Foundation and the Swiss Association of Cardiovascular Prevention and Rehabilitation support the project through advertisement of the InterHerz study on their website and through the distribution of flyers. Assuming more than 2,000 patients will be informed about our web-based intervention, we expect about 300 patients to show initial interest via email. According to our earlier research on web-based interventions, about half of the patients who initially show interest and contact us will give their written consent and fill out the screening questionnaires (n = 150). After a clinical interview and the application of inclusion criteria we expect a total number of 80 patients to be randomized to the two treatment conditions (for details see the flow chart in Figure 1).

### Inclusion and exclusion criteria

Adults who suffer from any cardiovascular disorder (including coronary heart disease, chronic heart failure, and arrhythmias) can apply to participate. Patients are included if they return the informed consent, are 18 years or older, have symptoms of depression (as defined by scoring above a cut-off of 9 on the Beck Depression Inventory (BDI-II)), are of sufficient knowledge of German language, and have access to the Internet. If patients have any acute health threatening disease (for example, cancer), a severe psychiatric illness (for example, psychosis, dementia), suicidal thoughts or plans, or an unstable heart condition, they are excluded from the study. Participants will remain in the study in case of further progression of heart disease during study participation.

### Intervention

The intervention is a web-based psychological treatment called Deprexis, which has been evaluated in previous trials [8] (http://www.deprexis.de). Deprexis was developed to reduce depressive symptoms in patients who do not have any somatic disease. The treatment consists of 10 content modules. All modules can each be completed in 10 to 60 minutes, depending on the user’s reading speed, interest, motivation, and individual path through the program. The modules are organized as simulated dialogues in which the program explains and illustrates concepts and techniques, engages the user in exercises, and continuously asks users to respond by selecting from response options. Subsequent content is then tailored to the users’ responses, resulting in a simulated conversational flow. The modules cover a variety of therapeutic content that is broadly consistent with a cognitive-behavioral perspective, although the program is not restricted to one cognitive behavioral therapy (CBT) manual. Instead, an effort was made to design the program as an integrative treatment tool that provides a variety of relevant therapeutic approaches and fits within the broad array of contemporary CBT (see Table 1). The link to the website of the InterHerz study is provided from the applicant’s institution (https://www.online-therapy.ch/interherz).

**Table 1:** Components included in the psychological treatment Deprexis

**Number**	**Component**
1	Behavioral activation
2	Cognitive modification
3	Mindfulness and acceptance
4	Interpersonal skills
5	Relaxation, physical exercise and lifestyle modification
6	Problem solving
7	Childhood experiences and early schemas
8	Positive psychology interventions
9	Dreamwork and emotion-focused interventions
10	Psychoeducation

During their work through Deprexis, participants are guided by a clinical psychologist. Weekly feedback on patient’s work and progress are given via an integrated email system. Participants are able to contact the psychologist via email if they have any questions during the intervention period.

### Assessment

The efficacy of the intervention is assessed post-treatment (T2) and additionally at 6 months follow-up (T3). Pre-treatment assessment (T0) is conducted by a psychologist before the randomization procedure to assess the level of depression, and to collect information on perceived stress, perceived social support and quality of life. At baseline, we further assess medical information on the disease, duration of the disease, time of the event, risk behavior (for example, smoking, low physical activity, poor diet), and medication or other psychotherapeutic treatments. Moreover, a clinical diagnostic interview is conducted by phone. To address the research question, depression levels are measured during treatment (T1), and treatment satisfaction is measured at the end of the treatment (T2).

### Measures

The German version of the Beck Depression Inventory (BDI-II) [40] is administered to assess the level of depressive symptoms. Ten points or more is to be considered a clinically relevant score for depressive symptomatology. All patients with depressive symptoms above this cut off are asked to fill out the German version of the Patient Health Questionnaire (PHQ-D) [41] and are additionally interviewed by a psychologist with the Structured Clinical Interview for DSM diagnosis (SCID) to classify patients as clinically depressed according to the American Psychiatric Association Diagnostic and Statistical Manual [42]. Stress perception is assessed by the Perceived Stress Questionnaire PSQ [43] and perceived social support by the German version of the self-rated 7-item ENRICHD Social Support Inventory (ESSI) [44]. The World Health Organization (WHO) quality of life-BREF (WHOQOL-BREF) (German version) [45], a brief form of a transcultural quality of life assessment instrument developed by the WHO, is used to measure quality of life. Client satisfaction is assessed post-treatment with the German version of the Client Satisfaction Questionnaire (CSQ) [46]. Medication and compliance with medication are assessed by free text and health-related lifestyle (for example smoking, physical activity, diet) with dichotomous items in line with earlier studies by the research team [47].

### Ethics considerations

The study protocol was approved by the Local Ethical Committee of the Faculty of Human Sciences, University of Bern and is in accordance with the Declaration of Helsinki. All patients receive written information about the aim of the study, benefits and risks of participation and the exact study procedure before giving their written informed consent to participate in the study. At that time all participants are informed that they can cancel participation without disclosing any reason at any time during the study.

With each participant an individual emergency plan is developed at the beginning of the study via a personal call. They receive information on nearby facilities in case of an emotional crisis (crisis intervention ward, psychiatric emergency units). In addition, an emergency link on the homepage provides telephone numbers and several addresses of treatment institutions that can be contacted 24 h a day. Patients with a suicidal tendency are excluded from the study. They are informed that a web-based intervention is not an appropriate treatment for patients in emotional crisis and are asked to seek help within a face-to-face setting. We assist patients in search of these settings.

### Statistical analyses

We will analyse the longitudinal data according to a completer analysis (after the intervention) and in addition we will use intent-to-treat analysis including patients who drop out during the intervention or waiting period. The efficacy of the intervention will be estimated using analysis of variance with baseline values as covariates and additional assessments conducted after 5 sessions (midterm) and after 10 sessions (post). The primary endpoint will be depressive symptoms measured by BDI II and a diagnostic interview after 10 weeks. Secondary end points will be perception of stress, social support, quality of life and compliance. Long-term stability of the efficacy of the intervention will be analysed by comparisons of pre-treatment and follow-up assessments of depression in all participants (InterHerz and waitlist control group) using the *t*-test of dependent samples, and pre-treatment to follow-up effect sizes will be calculated. For the prediction of treatment success we will use clinical variables (for example, severity of cardiac disease), sociodemographic variables (for example, age, gender) and process measures (for example, working alliance). Since missing values are a critical issue in longitudinal studies we will use a statistical algorithm for the estimation of missing values as implemented in SPSS.

## Discussion

This study examines the efficacy of a web-based, guided self-help treatment for depressed cardiac patients in terms of depressive symptoms, perception of stress, social support, quality of life and health behavior change. So far, no randomized controlled trial exists that specifically evaluates the efficacy of a web-based psychological treatment for cardiac patients with depressive symptoms. Given the fact that cardiac patients have limited access to psychological treatments, it is important to know whether such an easily accessible and low-cost intervention has future potential to be an integrative part in the rehabilitation of cardiac patients and in the general health system.

As patients with heart disease will be on average a relatively old patient group, we asked patients in a cardiac rehabilitation unit about the use of the Internet for health-related information as part of our preparation of the study protocol. In a sample of 59 patients (mean age 67 yrs, SD 8.6) 71% of the cardiac patients reported that they had their own access to the Internet and would use it as a source for health-relevant information on a regular basis. Furthermore, over 80% expressed interest in using a new web-based intervention. Whether these patients would benefit from a web-based psychological treatment specifically developed for depression remains unclear.

There are limitations to the study design. First, the intervention is not specifically adapted for cardiac patients and the effect on cardiac health will not be in the main focus of the investigation. Second, participants of the waitlist receive access to the psychological treatment after a delay of ten weeks, therefore, we will not be able to compare groups at the 6-month follow-up.

The study has several strengths. It has a high external validity since patients are recruited from cardiology institutions and organizations, and since patients suffering from various cardiovascular disorders are included. Depression is assessed by self-rated instruments and a diagnostic interview. This allows more profound knowledge of the actual mental state of the participant. Furthermore, to our knowledge this is the first web-based psychological treatment for cardiac patients using a proven web-based depression tool. This investigation will allow us to extend our knowledge of the benefits and limits of a web-based psychological treatment that integrates the most recent treatment approaches for depressed cardiac patients.

## Trial status

Recruitment of participants is ongoing; it began in March 2012 and is expected to end in June 2013.

## Abbreviations

BDI II: Beck Depression Inventory; CBT: Cognitive behavioral therapy; CREATE: Canadian cardiac randomized evaluation of antidepressant and psychotherapy efficacy; CSQ: Client Satisfaction Questionnaire; ENRICHD: Enhancing recovery in coronary heart disease patients with depression; ES: Effect size; ESSI: ENRICHD Social Support Inventory; IPT: Interpersonal psychotherapy; PHQ: Patient Health Questionnaire; PSQ: Perceived Stress Questionnaire; SCID: Structured Clinical Interview for DSM disorders; WHO: World Health Organisation; WHOQOL-BREF: WHO quality of life-BREF.

## Competing interests

The authors declare that they have no competing interests.

## Authors’ contributions

Principal responsibility for study design and conduct and project management is assumed by NMB. NMB, JB, and TB contributed to the development of the study design and data collection. NMB drafted the manuscript. All authors read and commented on drafts and approved the final manuscript. GAIA AG, the developer and distributor of Deprexis, neither interfered with the design of the trial nor provided direct support (for example, subject recruitment), except for the delivery of free vouchers for Deprexis. In case of technical questions, participants could turn to the online GAIA help desk. Staff, however, did not inquire or comment on the study.
